# Numerical Simulation and Experimental Study on Picosecond Laser Polishing of 4H-SiC Wafer

**DOI:** 10.3390/mi16101163

**Published:** 2025-10-14

**Authors:** Yixiong Yan, Yuxuan Cheng, Sijia Chen, Yu Tang, Fan Zhang, Piaopiao Gao

**Affiliations:** 1 College of Mechanical and Vehicle Engineering, Changsha University of Science & Technology, Changsha 410114, China; 2 State Key Laboratory of Precision Manufacturing for Extreme Service Performance, School of Automation, Central South University, Changsha 410083, China; 3 School of Intelligent Manufacturing and Energy Engineering, Zhejiang University of Science and Technology, Hangzhou 310023, China

**Keywords:** silicon carbide wafer, picosecond laser, polishing, surface quality, two-temperature model

## Abstract

4H-SiC wafers usually require polishing treatment after slicing to improve the surface quality. However, traditional polishing processes have problems such as low removal efficiency and easy surface damage, which affect the reliability of electronic devices. In this paper, picosecond laser polishing technology is used to study the 4H-SiC wafers after slicing. Numerical models of single-pulse ablation and moving heat source polishing were established to reveal the interaction mechanism between laser and material, including the dynamic evolution of free electron density and the remarkable spatiotemporal non-equilibrium heat transfer characteristics of the electron–lattice system. The sliced 4H-SiC surface with a roughness of 2265 nm was polished by a 1064 nm picosecond laser, and the influence of laser power and scanning speed on the surface quality was systematically studied. By collaboratively optimizing the polishing power and speed, the surface roughness of the sample can be significantly reduced to 207.33 nm (a decrease of 90.85%). The research results indicate that an ultrafast laser is suitable for the pretreatment process of sliced silicon carbide wafers, laying a foundation for further research in the future. This research has a certain research significance for promoting the development of ultrafast laser polishing technology for single crystal silicon carbide wafers and improving the performance and reliability of semiconductor devices.

## 1. Introduction

As one of the third generation of wideband gap semiconductor materials, 4H-SiC has excellent physical and chemical properties such as high hardness [1], high thermal conductivity [2], and high breakdown electric field [3], and has key application value in the fields of high-temperature, high-frequency, and high-power electronic devices [4,5]. However, in the SiC wafer manufacturing process, the slicing process often increases the surface roughness of the material [6] and causes microscopic defects such as cleavage cracks [7]. These defects not only reduce the electrical performance of the device but also affect the reliability of subsequent thin film deposition and device integration [8]. Therefore, it is necessary to reduce the surface roughness, eliminate subsurface damage, and improve the surface quality of the wafer through polishing processes.

Traditional wafer polishing methods, such as mechanical polishing and chemical/mechanical polishing, have obvious limitations in 4H-SiC processing. Mechanical polishing is prone to introducing subsurface damage and microcracks, leading to a decrease in material strength [9]. Chemical/mechanical polishing techniques, on the other hand, have low material removal rates due to the high hardness and chemical inertness of the materials [10,11], and it is difficult to balance the improvement of efficiency with the suppression of subsurface damage [12]. In addition, the chemical slurry used in the process introduces serious waste contamination issues [13,14]. In addition, these methods struggle to achieve local selective polishing and have limited effects on complex surface topography. Ultrafast lasers have gradually been applied in polishing processes due to their advantages such as high peak power, non-contact processing, and easy precise control [15,16]. Ultrafast laser polishing is fundamentally different from traditional mechanical polishing, chemical mechanical polishing, or continuous laser polishing. It mainly relies on the nonlinear absorption of laser energy by materials to achieve material ablation removal, and has significant advantages in controlling subsurface damage, achieving clean production, and enhancing process controllability [17,18].

Ultrafast laser polishing is currently mainly applied to metal materials such as stainless steel and titanium alloys. Chen et al. used a picosecond laser to polish ASP23 steel in a nitrogen environment and explored the influence of energy density and scanning strategy. The results showed that picosecond laser polishing can effectively reduce surface roughness and significantly lower the thermal deformation of workpieces [19]. Liu et al. explored the flow behavior and morphology evolution mechanism of the molten pool on the Cr12MoV surface by combining a nanosecond pulsed laser polishing experiment with a 3D computational fluid dynamics numerical simulation, which provided theoretical insights for the process optimization of high-energy pulsed laser polishing metal [20]. Le et al. established a nanosecond pulsed laser polishing model for SKD11 steel based on surface roughness and conducted experimental verification. Research showed that the influence of peak power on polishing efficiency was significantly stronger than that of pulse overlap rate. The error between the numerical model and the experimental results was less than 15%, providing a reliable parameter optimization basis for nanosecond laser precision polishing of die steel [21]. Li et al. proposed an ultrasonic-assisted underwater polishing method, using nanosecond pulsed lasers to polish TA15 titanium alloy. The cavitation effect induced by ultrasonic vibration promotes the flow of the molten pool, refines the grains, and inhibits oxidation, thereby significantly improving the surface quality and comprehensive mechanical properties of the polished surface [22].

In addition to metals, ultrafast laser polishing is also applicable to non-metallic materials such as glass and ceramics. Zhang et al. polished alumina ceramics using a picosecond laser overlapping parallel line scanning mode, achieving a surface roughness reduction from 1.80 μm to 0.32 μm. The research suggests that the mechanism of picosecond laser polishing lies in the remelting and recrystallization process of nanoparticles, which induces the formation of a dense fine-grained structure, thereby reducing the roughness [23]. Yang et al. calculated the single-pulse ablation threshold of RB-SiC based on the two-temperature model theory. Through experiments, the surface roughness Ra of RB-SiC was significantly reduced from approximately 200 nm to 43 nm, and the polishing efficiency was increased by 72% compared with traditional mechanical polishing. This study verified the feasibility of femtosecond laser polishing, indicating that it can replace some intermediate processes of traditional mechanical polishing [24]. Zhang et al. investigated the effects of infrared femtosecond laser pulse energy and defocus amount on the surface morphology, roughness, and oxidation degree of SiC ceramic polishing. The surface microstructure and elemental changes under different parameters were analyzed, providing a reference for the optimization of process parameters of femtosecond laser polishing of SiC ceramics [25]. Although ultrafast laser polishing technology has demonstrated significant potential and accumulated rich research results in the surface treatment of various metal and non-metal materials, research focusing on ultrafast laser polishing of single crystal 4H-SiC is relatively scarce and still needs further exploration at present.

In this paper, numerical models of single-pulse ablation and moving heat source polishing for picosecond laser polishing of 4H-SiC wafers were established. The variation laws of free electron density and temperature were simulated, revealing the mechanism of interaction between picosecond lasers and materials. The influence of laser power and scanning speed on surface quality was analyzed through experiments. The material evolution mechanism in the laser polishing process was explored by microscopic morphology observation and elemental composition analysis. Through a systematic analysis of simulations and experiments, it has been demonstrated that ultrafast lasers are suitable for the pre-polishing process of sliced silicon carbide wafers, laying the foundation for further research in the future.

## 2. Numerical Model

### 2.1. Two-Temperature Model

When a picosecond laser is focused on the 4H-SiC surface, electrons absorb photon energy and transition to form free electrons. The evolution of free electron density can be expressed by the Fokker–Planck equation [26]:

(1) $$\frac{\partial n_{e}}{\partial t}=D\nabla^{2}n_{e}+\frac{\alpha I}{\hbar \nu }+\frac{\beta I^{2}}{2\hbar \nu }-An_{e}-B{n_{e}}^{2}-C{n_{e}}^{3}$$

where n_e_ is the electron density, α is the single-photon absorption coefficient, β is the two-photon absorption coefficient, ℏ is the reduced Planck constant, A is the single-photon recombination coefficient, B is the two-photon recombination coefficient, C is the Auger recombination coefficient, and I is the laser intensity.

Free electrons collide during acceleration and the temperature rises dramatically. The heated electrons interact with phonons in the lattice and transfer energy to the lattice. The two-temperature model describes the variation of the electron temperature and the lattice temperature in time and space, which is expressed by two coupled partial differential equations [27]:

(2) $$C_{e}\frac{\partial T_{e}}{\partial t}=\nabla \left(k_{e}\nabla T_{e}\right)-G\left(T_{e}-T_{l}\right)+S\left(r,z,t\right)$$

(3) $$C_{l}\frac{\partial T_{l}}{\partial t}=\nabla \left(k_{l}\nabla T_{l}\right)+G\left(T_{e}-T_{l}\right)$$

where T_e_ and T_l_ are the electron and lattice temperatures, respectively; S is the laser heat source absorbed by the material; and G is the electron/phonon coupling constant. The volume heat source S (r, z, t) is related to the intensity decay of the laser light within the crystal, which follows the Beer–Lambert law and is expressed as follows:

(4) $$S\left(r,z,t\right)=I\left(r,t\right)\alpha \exp\left(-\alpha z\right)$$

where I (r, t) is the laser intensity and z is the depth of penetration down the surface. The laser intensity I (r, t) is expressed in terms of the peak power density and conforms to a Gaussian distribution in time and space:

(5) $$I\left(r,t\right)=\frac{P}{f\pi \omega_{0}^{2}}\frac{1-R}{t_{p}}\sqrt{\frac{4\ln2}{\pi }}\exp\left(-4\ln2\frac{{\left(t-t_{p}\right)}^{2}}{t_{p}^{2}}\right)\exp\left(-\frac{2r^{2}}{\omega_{0}^{2}}\right)$$

where P is the average laser power, f is the repetition frequency, ω_0_ is the beam radius, R is the reflectivity, and t_p_ is the pulse width.

### 2.2. Material Removal Simulation

The essence of picosecond laser polishing lies in the fact that the energy deposition within an extremely short period of time causes the surface temperature of the material to rise rapidly, inducing local phase transformation and thereby selectively ablating and removing the material. The ablation process was simulated using boundary heat flux and deformation geometry in COMSOL Multiphysics 6.3 software, assuming that 4H-SiC was directly transformed from the solid phase to the gas phase by a picosecond laser. The boundary heat flux q_a_ required for ablation is

(6) $$q_{a}=h_{a}\left(T_{l}-T_{v}\right)$$

where h_a_ is the heat transfer coefficient and T_v_ is the vaporization temperature. The material removal is represented by the mesh deformation of the laser incident surface, and the normal mesh displacement velocity is calculated as

(7) $$v_{mesh}=\frac{q_{a}M}{\rho L_{v}}$$

where M is the molar mass, ρ is the density, and L_v_ is the latent heat of vaporization.

### 2.3. Axisymmetric Model of Laser Ablation

**Figure 1:**
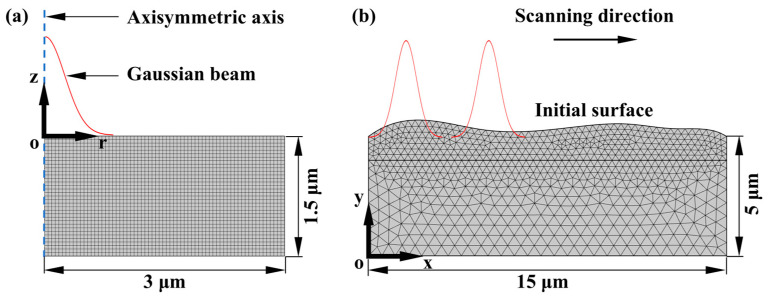
Geometric model and mesh: (**a**) 2D axisymmetric single-pulse ablation model; (**b**) 2D moving heat source polishing model.

Studying the variation laws of free electron density, electron temperature and lattice temperature under different parameters are of vital importance for understanding the energy transfer process and predicting the ablation depth, making it the foundation of laser polishing research. Therefore, a numerical model for the ablation of 4H-SiC by single pulse picosecond laser was established first. Due to the rotational symmetry of Gaussian beams, a two-dimensional axisymmetric model was established to simplify the calculation, as shown in Figure 1a. The center of the Gaussian beam coincided with the axis of symmetry z, and meshing was performed using a free quadrilateral mesh. The volume heat source acted on the entire domain, with the upper surface set as a heat convection boundary and the lower and right surfaces set as heat insulation boundaries.

### 2.4. Two-Dimensional Model of Laser Polishing

On the basis of analyzing the temperature field distribution and ablation profile using the single-pulse ablation model, a two-dimensional laser polishing numerical model with a picosecond laser as a moving heat source was established. The geometric model and meshing are shown in Figure 1b, where the laser acts on the rough surface and moves from left to right. The two-temperature equations and boundary condition settings remain unchanged. Due to the introduction of the laser scanning speed and the change in the coordinate system, the laser intensity and the heat source are represented by I (x, t) and S (x, y, t), respectively:

(8) $$I\left(x,t\right)=\frac{P}{f\pi \omega_{0}^{2}}\frac{1-R}{t_{p}}\sqrt{\frac{4\ln2}{\pi }}\exp\left(-4\ln2\frac{{\left(t-t_{p}\right)}^{2}}{t_{p}^{2}}\right)\exp\left(-\frac{2({x-vt)}^{2}}{\omega_{0}^{2}}\right)$$

(9) $$S\left(x,y,t\right)=I\left(x,t\right)\alpha \exp\left(-\alpha y\right)$$

### 2.5. Properties and Parameters

A series of simulations of single-pulse ablation and moving heat source polishing were performed to analyze the results at different laser powers and scanning speeds. The picosecond laser parameters and the thermophysical parameters of 4H-SiC are shown in Table 1 [28].

## 3. Experimental Setup

**Figure 2:**
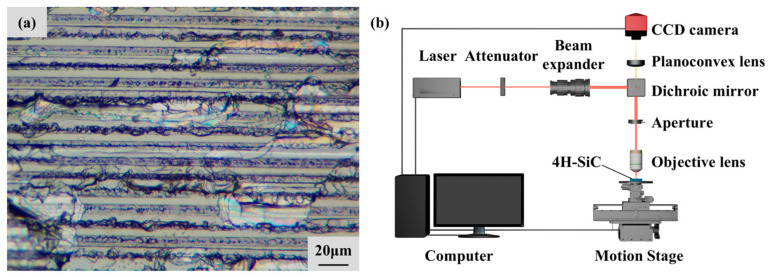
Experimental materials and devices: (**a**) surface morphology of the 4H-SiC sample to be polished; (**b**) laser polishing experimental setup.

The material used in this article is a 4H-SiC wafer produced by Shandong Tianyue Technology Co., Ltd. (Jinan, China). Before the laser polishing experiment, the wafer had been cut into a 2 cm × 2 cm sample and separated through a laser slicing process, as shown in Figure 2a. The initial surface roughness after slicing is approximately 2265 nm. The sliced sample was ultrasonically cleaned in anhydrous ethanol for 10 min. The sliced surface was used as the processed surface for laser polishing experiments, and all polishing experiments were completed on this sample.

The experimental setup is shown in Figure 2b. The infrared picosecond laser (BGL-1064-10B, BWT, Tianjin, China) output a laser with a wavelength of 1064 nm and a pulse width of 10 ps. The laser beam was focused on the sample surface through a beam transmission system composed of an adjustable attenuator, a beam expander, a dichroic mirror and a focusing objective lens. The laser focus was controlled by the *Z*-axis translation of the motion platform and underwent secondary verification through a CCD camera. The sample was placed on the carrier platform, and the XY axis of the platform was controlled for raster motion to achieve sample scanning. After the laser polishing experiment was completed, the appearance of the polished surface was observed using an optical microscope (TM17-3M180, AOSVI, Shenzhen, China). The overall topography and roughness of the polished surface were measured using an optical profilometer (Contour GT-K, Bruker, Karlsruhe, Germany). The microstructure features were characterized using a scanning electron microscope (SEM5000, CIQTEK, Hefei, China). The crystal structure and elemental composition changes in 4H-SiC before and after polishing were analyzed by EDS (X-MaxN 50, Oxford Instruments, Oxford, UK) and Raman spectrometer (DXR3, Thermo Scientific, Waltham, MA, USA).

## 4. Results and Discussion

### 4.1. Simulation Results

#### 4.1.1. Free Electron Density Evolution

The evolution of free electrons during the picosecond laser ablation of 4H-SiC clarifies the interaction mechanism between the laser and the material, which demonstrates the dynamic process of the material from absorbing laser energy to final ablation. Simulating the evolution of electron density is helpful in revealing the processes of multiphoton absorption, electron excitation, and energy relaxation.

**Figure 3:**
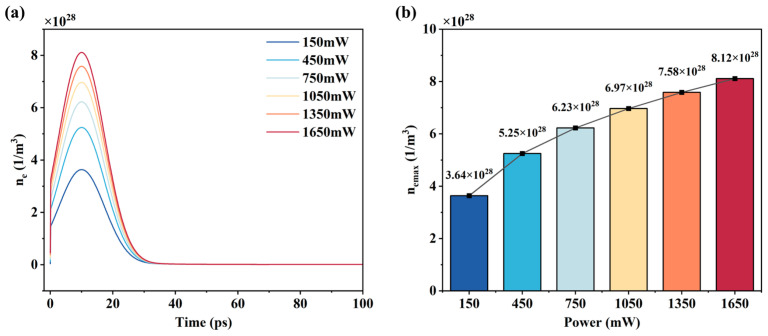
Simulation results of free electron density: (**a**) variation curves of free electron density at different powers; (**b**) variation of peak free electron density with laser power.

Figure 3a illustrates the results of free electron density with time at different powers. In the initial stage, the free electron density rapidly increased from the order of 10^17^ to 10^28^, and ultimately reached its peak at 10 ps. This is due to the high peak power density of the picosecond laser that causes multiphoton absorption in SiC, and the valence band electrons are excited to the conduction band to form electron/hole pairs. At the same time, the initial free electrons in the material are accelerated by the strong laser electric field, which triggers collisional ionization, leading to an exponential increase in the free electron density. After 10 ps, due to the transfer of energy from the hot electrons to the lattice via electron/phonon collisions, the free electron density on the surface of the material gradually decreases until it stabilizes after 30 ps.

Figure 3b shows the curve of the peak free electron density with power. With the increase in power, the laser electric field intensity increases, leading to the enhancement of the collisional ionization effect, which results in the growth of the free electron density. However, when the free electron density approaches the critical density, the plasma-shielding effect of the material becomes significant. Therefore, when the laser power continues to increase, the increment of electron excitation gradually decreases and eventually saturates.

#### 4.1.2. Electron and Lattice Temperature Evolutions

The variations in electron temperature and lattice temperature reflect the process of laser energy transfer between the electron and the lattice. Analyzing the variation curves of these two can reveal the propagation characteristics of laser energy in the material, the establishment process of thermal equilibrium, and the energy distribution features in the ablation process.

**Figure 4:**
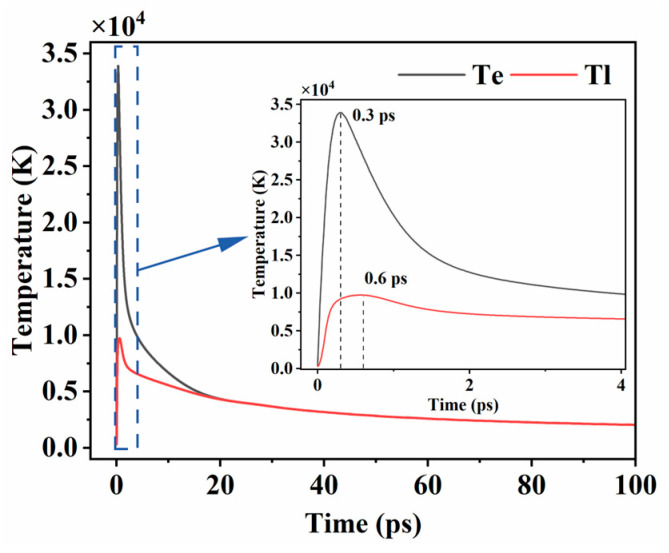
Electron temperature and lattice temperature variation curves at 750 mW power.

The evolution of the electron and lattice temperatures at the center point of the material surface (r = 0, z = 0) at a power of 750 mW was simulated using a single-pulse ablation model, and the results are shown in Figure 4. In the initial stage of laser action on the material, the electron and lattice systems are in a non-equilibrium state. Due to the low mass and heat capacity of the electrons, their temperature rises rapidly in a very short period of time. From the simulation results, the electron temperature reaches a peak of about 3.39 × 10^4^ K at 0.3 ps. At the same time, the electron transfers energy to the lattice through electron/phonon coupling. However, due to the higher mass and thermal conductivity of Si and C atoms in the lattice, the lattice temperature rise has a hysteresis, and the peak temperature is lower. The peak temperature is about 9.73 × 10^3^ K, which occurs at a moment of about 0.6 ps. As time passes, the electron and lattice temperatures gradually decrease, and finally the system reaches thermal equilibrium at a moment of about 20 ps.

**Figure 5:**
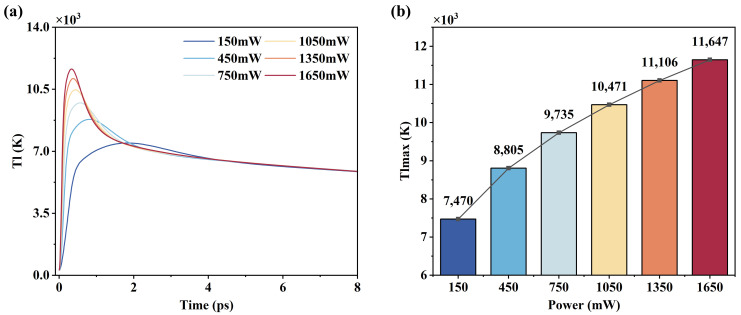
Simulation results of lattice temperature: (**a**) lattice temperature variation curves at different powers; (**b**) peak temperature variation with power.

The simulation results of lattice temperature evolution at different powers are shown in Figure 5a. As the laser power increases, the lattice temperature rises significantly faster and peaks in a shorter time. In addition, higher power intensities produce larger peak temperatures, and the temperature decreases faster. The trend of the peak temperature at different powers is shown in Figure 5b, where it can be seen that the increment of the peak temperature is gradually decreasing. This is because the plasma generated when the laser power is excessively high produces refraction, scattering, and other effects on the laser beam, resulting in a reduction in energy input. At the same time, a large amount of energy needs to be absorbed for material vaporization to occur, resulting in a limited further increase in lattice temperature.

#### 4.1.3. Single-Pulse Ablation

**Figure 6:**
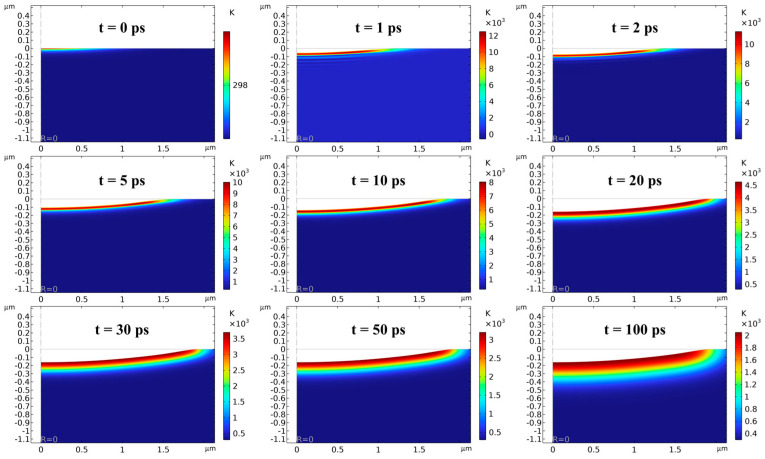
Single-pulse ablation profiles at different moments at 750 mW.

The single-pulse ablation simulation provides a theoretical basis for the selection of process parameters and prediction of material removal for subsequent laser polishing experiments by investigating the variation law of ablation pit diameter and depth with laser power. Figure 6 shows the lattice temperature distribution as well as the shape of the ablation profile inside the silicon carbide sample at different times when the laser power is 750 mW. In the time from t = 0 to 2 ps, the temperature distribution mainly reflects the rapid heating of the surface material, and ablation has not yet occurred significantly. After t = 2 ps, the high-temperature region gradually spreads to the interior of the material, and the profile of the ablation pit becomes more obvious, and its depth and diameter increase significantly. At around t = 30 ps, the maximum temperature inside the SiC is less than the vaporization temperature of 3840 K, and the profile of the ablation pit has basically stabilized. The main process at this time is that the heat inside the material continues to spread deeper and wider, and the temperature gradient around the ablation pit gradually decreases. With the extension of time, at t = 100 ps, an ablation pit with a depth of 160.60 nm and a diameter of 3.80 μm is formed on the SiC surface.

**Figure 7:**
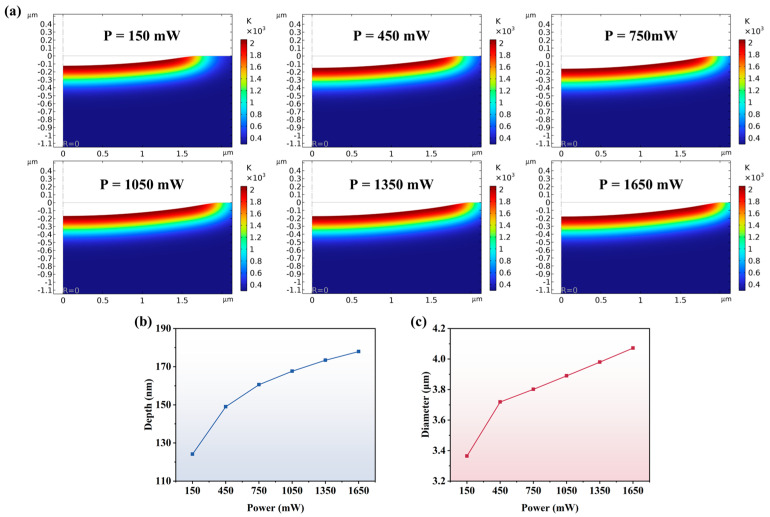
(**a**) Single-pulse ablation profile at different powers; (**b**) variation curve of ablation depth with laser power; (**c**) variation curve of ablation diameter with laser power.

Figure 7 further analyzes the profiles of the ablation pits and the temperature field distribution around them at different powers. As shown in Figure 7a, the heat-affected zone increases with increasing power, and the ablation pit depth and diameter also increase significantly. Figure 7b and Figure 7c represent the curves of ablation pit depth and ablation pit diameter with power, respectively. The ablation pit depth increases from 124.14 nm to 177.93 nm and the diameter from 3.36 μm to 4.07 μm when the power is increased from 150 mW to 1650 mW. By calculation, the ablation pit depth increased by 43.33%, which is greater than the diameter increase of 21.13%. This is due to the combination of the high energy distribution characteristics at the center of the Gaussian beam and the longitudinal penetration of the laser. In addition, the increment of ablation pit depth decreases gradually under the same power increment, which may be associated with the plasma-shielding effect as well as the change in the surface material properties during the ablation process.

#### 4.1.4. Multi-Pulse Polishing

**Figure 8:**
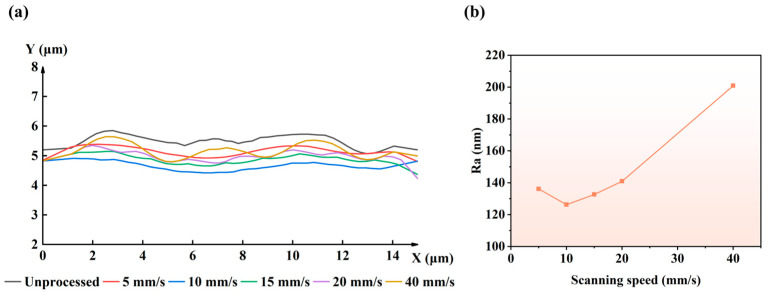
Simulation results of laser polishing at different scanning speeds: (**a**) comparison of polished surface profiles; (**b**) variation curve of surface roughness Ra.

The simulation results of 2D laser polishing with a picosecond laser as a moving heat source are shown in Figure 8. The laser power is 750 mW, and the scanning speed is 5, 10, 15, 20, and 40 mm/s. The surface profiles and surface roughness Ra are compared before and after polishing, and the results are shown in Figure 8a and Figure 8b, respectively. The roughness Ra of the unpolished surface is 167.32 nm, and the undulation of the surface profile curve is obvious. As the scanning speed increases, the roughness of the polished surface decreases and then increases. The lowest roughness is 126.29 nm at a scanning speed of 10 mm/s, and the surface profile is the flattest. When the scanning speed is between 10 and 40 mm/s, the surface roughness Ra increases gradually. This is because the laser pulse overlap rate decreases, the energy density deposited on the material surface decreases, and the material removal becomes less effective.

### 4.2. Experimental Results

During the laser polishing experiments, the laser repetition frequency and scanning interval were kept constant at 100 kHz and 2 μm, respectively. Two sets of single-factor experiments were set up to investigate the effects of laser power and scanning speed on the laser polishing quality. Based on the results of the single-pulse ablation simulation and the moving heat source polishing simulation, the ranges of laser power and scanning speed were determined, respectively. Firstly, the scanning speed was kept constant at 20 mm/s, and the laser power was set in the range of 750 to 1150 mW to polish the sliced surface. Subsequently, polishing experiments were conducted with an 850 mW laser at different speeds within 1 to 100 mm/s.

#### 4.2.1. Influence of Laser Power on Surface Roughness

**Figure 9:**
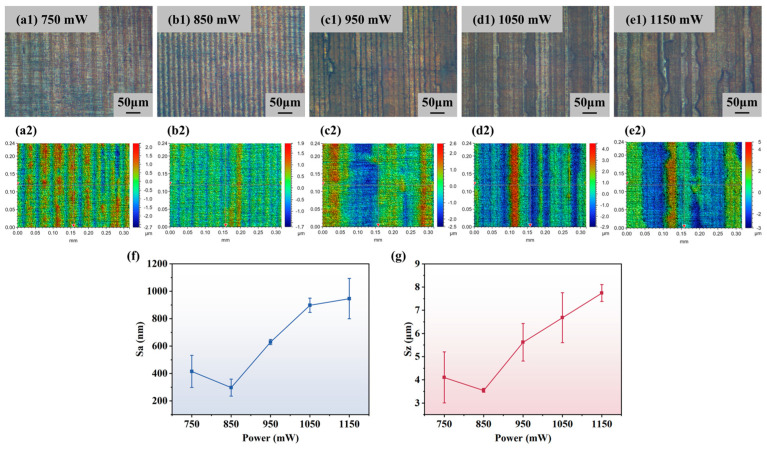
Effect of laser power on polishing effect: (**a1**–**e1**) images under optical microscope; (**a2**–**e2**) images under optical profilometer; (**f**) variation curve of surface roughness Sa with laser power; (**g**) variation curve of maximum height difference Sz with laser power.

The effect of laser power on the polished surface quality was first explored under the condition of a constant scanning speed of 20 mm/s. In order to fully achieve the polishing effect and avoid thermal damage to the material caused by excessive power, combining with the simulation results, the laser power was set to 750, 850, 950, 1050, and 1150 mW. Figure 9 shows images of the polished sample surfaces under the optical microscope as well as the results of the roughness tests. As seen in Figure 9(a1–e1), the colors of the sample surfaces after polishing are all darkened and different degrees of ablation appear, which is more obvious when the power is greater than 950 mW. Figure 9(a2–e2) show the images under the optical profilometer. It can be clearly seen that among the five sets of results, the polished surface under 850 mW power is the flattest. At 750 mW, the initial surface bumps were not fully polished due to the lower laser power. For the three sets of samples at higher laser power shown in Figure 9(c2–e2), the surface morphology deteriorated. This may be attributed to the concentration of defects in the initial surface gully edge region, where more energy is absorbed at higher power, leading to more severe surface ablation. The average surface roughness Sa variation curve with power is shown in Figure 9f. At 850 mW, the minimum surface roughness is 297.59 nm, and the roughness gradually increases with the increase in power, reaching a maximum value of 946.33 nm at 1150 mW. The variation trend of the maximum surface height difference, Sz, is similar to that of Sa with the increase in power, which also shows a trend of decreasing first and increasing later. As shown in Figure 9g, the minimum value of Sz, namely 3.55 μm, appeared at 850 mW power. This is mainly attributed to the optimal balance between effective material removal and thermal damage suppression. At this power, the laser energy density is sufficient to efficiently remove micro protrusions on the sliced surface through ablation, achieving surface leveling while avoiding significant negative effects caused by high power. The simulation results show that when the power exceeds a certain range, the increase in lattice peak temperature tends to saturate, and excessive heat input can cause severe material melting, gasification, and splashing, leading to defects such as recast layers and micro pits, thereby deteriorating the surface morphology. Therefore, 850 mW is a key optimization process window that can achieve effective polishing while minimizing thermal damage.

#### 4.2.2. Influence of Scanning Speed on Surface Roughness

**Figure 10:**
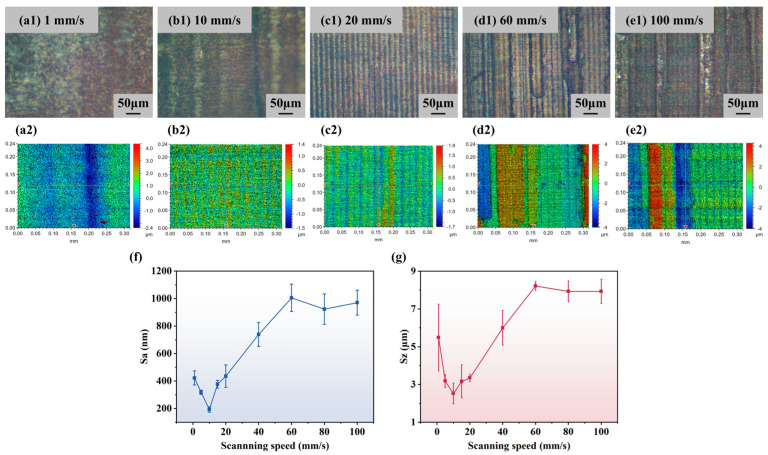
Effect of scanning speed on polishing effect: (**a1**–**e1**) images under optical microscope; (**a2**–**e2**) images under optical profilometer; (**f**) variation curve of surface roughness Sa with scanning speed; (**g**) variation curve of maximum height difference Sz with scanning speed.

In addition to the laser power, this paper also explores the effect of scanning speed on the polished surface quality. The polishing experiments were carried out at a power of 850 mW with scanning speeds set to 1, 5, 10, 15, 20, 40, 60, 80, and 100 mm/s, and the results are shown in Figure 10. Figure 10(a1–e1) show images of the polished samples under an optical microscope at scanning speeds of 1, 10, 20, 60, and 100 mm/s, and the corresponding Figure 10(a2–e2) show the images under an optical profilometer. It can be seen that the surface of the polished samples showed different degrees of dark ablation marks. At a scanning speed of 1 mm/s, the ablation marks on the surface are obvious and the roughness is large. When the speed is increased to 10 mm/s, the polished surface is the most uniform and has the lowest roughness. However, the higher the scanning speed, the lower the laser energy density, and the more difficult it is to remove the grooves on the original surface. Therefore, as the scanning speed continues to increase, the uniformity of the polished surface gradually becomes worse.

The variation curves of surface roughness Sa and maximum height difference Sz with scanning speed are shown in Figure 10f,g. Overall, the polished surface quality is best at 10 mm/s, and both Sa and Sz show a trend of decreasing and then increasing. When the scanning speed is less than 10 mm/s, the excessive heat accumulation leads to violent material vaporization and melt ejection, and the cooling and solidification form irregular recast layers and micro-pits, so the polished surface quality is poor. When the scanning speed is 10 mm/s, the energy deposition rate and the thermal diffusion rate of the material basically reach the equilibrium state, and the polishing effect is optimal, at which Sa is 207.33 nm. When the scanning speed is between 10 and 60 mm/s, both Sa and Sz show a rapid increasing trend, which is caused by the gradual decrease in energy input. When the scanning speed is greater than 60 mm/s, Sa and Sz basically remain stable.

**Figure 11:**
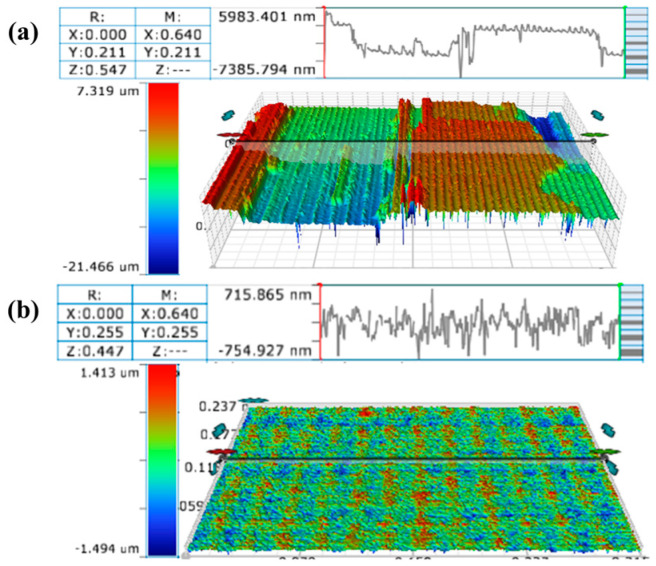
Comparison of 3D morphology of the sample surface before and after polishing: (**a**) original surface; (**b**) 850 mW, 10 mm/s polished surface.

The three-dimensional morphology of the sample surface polished under the parameters of 850 mW and 10 mm/s was compared with that of the original surface, as shown in Figure 11. The original large-area faults on the sample surface were transformed into a more uniform and flat morphology after polishing. The surface roughness Sa and the maximum height difference Sz of the sample decreased by 90.85% and 89.90%, respectively. Based on the measurements and calculations, the material removal rate of the method used in the experiments is about 3 μm/h. Higher material removal rates can be achieved compared to the conventional CMP methods [29,30,31], such as 0.74 μm/h for Fenton-Like Reaction polishing [32] and 0.694 μm/h for the photocatalysis-assisted CMP [33]. From the experimental results, the optimal laser scanning speed to achieve the best surface roughness of the sample is 10 mm/s, which is consistent with the results of the polishing model. The synergistic effect between simulation and experiment indicates that 10 mm/s provides an ideal pulse overlap rate for effective material removal without excessive heat accumulation. Picosecond laser polishing has not yet reached the precision required for industrial grade polishing, mainly due to the dominant thermal mechanism of laser action, which triggers uncontrollable phase transitions and microstructural changes. In future research, shorter pulse width/wavelength lasers can be used to suppress thermal effects and optimize beam and scanning strategies simultaneously to ensure uniform energy distribution.

#### 4.2.3. Micro-Morphology Analysis

**Figure 12:**
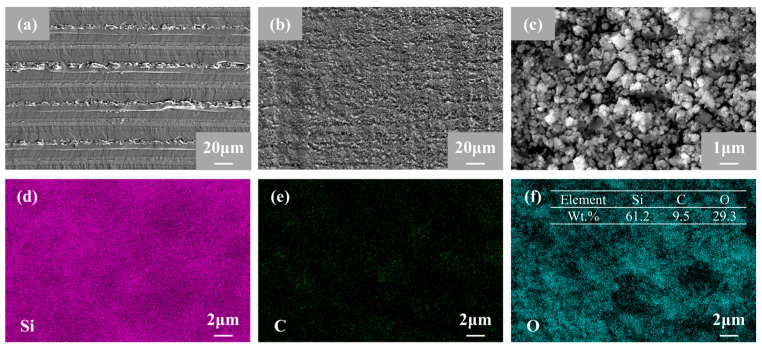
Microscopic morphology and elemental distribution of 850 mW and 10 mm/s polished samples: (**a**) SEM image of the original surface; (**b**) SEM image of the polished surface; (**c**) microstructure of the polished surface; (**d**) distribution of Si element; (**e**) distribution of C element; (**f**) distribution of O element.

In order to analyze the change in the micro-morphology of 4H-SiC before and after laser polishing, the surface of the samples at 850 mW and 10 mm/s was characterized by SEM, while the elemental distribution was detected using EDS, and the results are shown in Figure 12. Figure 12a shows the transverse modification lines and longitudinal disintegration cracks generated in the previous laser slicing process. After laser polishing, the surface of the sample showed a relatively flat condition without large-size defects, as shown in Figure 12b. The magnification was further enlarged to observe the microstructure, shown in Figure 12c. The material surface formed irregularly arranged nano-sized particles after polishing, and the particles accumulated to form micrometer-scale clusters and pores. The distribution of Si, C, and O elements is represented in Figure 12d–f, respectively. The C element on the surface of the material decreases dramatically after polishing, while the O element is introduced at the same time. Altogether, the material was thermally decomposed after absorbing the laser energy, and the particles were formed by cooling and solidification in a very short time, during which some of the material was oxidized. The formation of a surface oxide layer effectively reduces material hardness and promotes chemical reactions in the conventional CMP process, resulting in improved polishing efficiency, which suggests that ultrafast lasers are suitable for pre-processing silicon carbide wafers after slicing [34].

#### 4.2.4. Component Analysis

**Figure 13:**
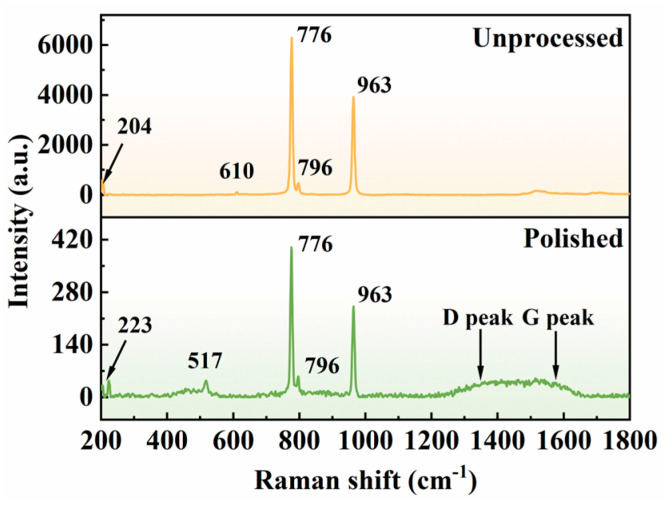
Comparison of Raman spectroscopy detection results before and after polishing.

To further examine the alterations in material composition induced by laser polishing, Raman spectroscopy was conducted on both the original and polished samples using a 532 nm laser source. The corresponding spectra are presented in Figure 13. The Raman profile of the original 4H-SiC exhibits characteristic peaks at 204 cm^−1^, 610 cm^−1^, 776 cm^−1^, 796 cm^−1^, and 963 cm^−1^, which correspond to five distinct phonon modes: E2(TA), A1(LA), E2(TO), E1(TO), and A1(LO), respectively. In the polished samples, sharp peaks remain observable at 776 cm^−1^ and 796 cm^−1^, though with markedly diminished intensity, suggesting a reduced presence of residual SiC crystals. Additionally, peaks emerging at 223 cm^−1^ and 517 cm^−1^ are ascribed to the formation of crystalline silicon. The broad spectral features within the range of 440–490 cm^−1^ are associated with amorphous silica and amorphous silicon. Furthermore, a broad band appears between 1200 and 1700 cm^−1^, attributed to the deposition of amorphous carbon, within which the broad peaks centered at 1350 cm^−1^ and 1580 cm^−1^ correspond to the D and G peaks, respectively. From the above results, the picosecond laser high-energy pulses caused thermal decomposition of the 4H-SiC surface, resulting in the formation of free Si and C. Some of the Si elements reacted with airborne O elements to form silica, while the unoxidized Si formed nanocrystalline or amorphous silicon under rapid cooling. Most of the C elements escaped as CO_2_ or CO gas, and the remaining portion was deposited in the form of graphitization on the surface of the samples.

## 5. Conclusions

In this paper, the process of picosecond pulsed laser removal of 4H-SiC surface material for polishing was simulated by establishing a single-pulse ablation model and a moving heat source model. Polishing experiments were carried out on 4H-SiC after the laser slicing process using a picosecond laser with a wavelength of 1064 nm, and the influence laws of laser power and scanning speed on the polished surface quality were discussed. The material evolution mechanism during the laser polishing process was explored by micro-morphology observation and elemental composition analysis. The 4H-SiC sliced surface with an initial surface roughness of about 2265 nm was polished under the process parameters of 850 mW and 10 mm/s, and the final surface roughness was reduced to 207.33 nm. The results of this study demonstrate the following:

(1) The two-temperature model reveals the dynamic process of the interaction between the picosecond laser and 4H-SiC. The evolution of free electron density is jointly regulated by multiphoton absorption and plasma-shielding effects, and the electron and lattice temperatures exhibit significant spatiotemporal non-equilibrium characteristics.
(2) The synergistic regulation of laser power and scanning speed is the key to determining the quality of 4H-SiC surface polishing. Excessive laser power will induce thermal damage and deteriorate the material surface, while insufficient power cannot effectively remove the material. The scanning speed significantly affects the polishing effect by regulating the energy density. Low speed induces thermal accumulation, leading to recast layer defects, while high speed reduces the material removal efficiency due to insufficient energy input.
(3) 4H-SiC underwent thermal decomposition, oxidation, and amorphization phase transition during picosecond laser polishing, and finally formed a multiphase hybrid system. The rapid cooling process after laser action resulted in the formation of the nanoparticle cluster/pore composite structure on the polished surface.
(4) This study has certain limitations, such as insufficient exploration of the process of suppressing material phase transition and the inability to achieve industrial grade surface quality on the surface at present. However, this study verified the technical feasibility of efficient planarization of rough surfaces of 4H-SiC using ultrafast lasers from both theoretical and experimental perspectives, providing a theoretical basis for its potential application as a pre-polishing process after slicing.

## Figures and Tables

**Table 1:** Laser parameters and thermophysical parameters.

Parameter Type	Parameter	Value
Laser parameters	Laser wavelength (λ)	1064 nm
	Laser frequency (ƒ)	100 kHz
	Beam waist radius (ω_0_)	1 μm
	Pulse width (t_p_)	10 ps
	Reflectivity (R)	0.5
Thermophysical parameters	Ambipolar diffusion coefficient (D)	2.5 cm^2^/s
	Single-photon absorption coefficient (α)	4.464 × 10^5^ 1/cm
	Two-photon absorption coefficient (β)	0.4 × 10^−11^ m/W
	Reduced Planck constant (ℏ)	1.055 × 10^−34^ J·s
	Single photon recombination coefficient (A)	3.846 × 10^6^ 1/s
	Two-photon recombination coefficient (B)	3 × 10^−11^ cm^3^/s
	Auger recombination coefficient (C)	7 × 10^−31^ cm^6^/s
	Initial electron concentration (n_e0_)	5 × 10^17^ 1/cm^3^
	Electron heat capacity (C_e_)	311.53 J/kg/K
	Electron thermal conductivity (k_e_)	2 × 10^−4^ W/m/K
	Electron/phonon coupling constant (G)	9.8 × 10^18^ W/m^3^/K
	Lattice heat capacity (C_l_)	690 J/kg/K
	Lattice thermal conductivity (k_l_)	370 W/m/K
	Material density (ρ)	3210 kg/m^3^
	Vaporization temperature (T_v_)	3840 K
	Latent heat of vaporization (L_v_)	4 × 10^5^ J/mol
	Molar mass (M)	4 × 10^−2^ kg/mol

## Data Availability

The data of this study are available from the corresponding author upon reasonable request.
